# Stress Fracture Risk in Military Trainees Is Associated With Modifiable Lifestyle and Training Factors: A Retrospective Case-Control Study

**DOI:** 10.7759/cureus.115226

**Published:** 2026-08-26

**Authors:** Laith Hseinat, Abdul-Rahman Al-Mwajeh, Naser Shari, Saif Al Zoubi, Omar Al Qudah, Malek Ghnaimat, Mohammad Al Bakri, Malek Abu Hazim, Mohammad Al Dweeri, Ashraf Albadaineh

**Affiliations:** 1 Department of Orthopaedics, Royal Medical Services, Amman, JOR; 2 Medical School, Jordan University of Science and Technology, Amman, JOR; 3 Department of Orthopaedics and Traumatology, Royal Medical Services, Amman, JOR; 4 Department of Orthopaedics and Traumatology, King Hussein Medical Center, Amman, JOR; 5 Department of Orthopaedics and Sport Medicine, King Hussein Medical Center, Amman, JOR; 6 Department of Orthopedics and Sport Medicine, Jordan Hospital, Amman, JOR; 7 Department of Orthopaedics and Traumatology, Jordanian Ministry of Health, Amman, JOR

**Keywords:** military trainees, retrospective case-control study, risk factors, stress fractures, vitamin d deficiency

## Abstract

Introduction/Objectives

Stress fractures are a common and debilitating injury in military populations. Early identification of risk factors is critical for prevention. The purpose of this study was to identify demographic, clinical, lifestyle, and training-related factors associated with stress fractures in military trainees and to characterize their anatomical distribution.

Methods

A retrospective case-control study was conducted at King Hussein Medical City from January 2021 to June 2023, including 600 military trainees (139 with stress fractures and 461 controls). Data collected included age, gender, training duration, prior conditioning, smoking status, vitamin D and calcium levels, previous joint injuries, and footwear usage. Statistical analysis was used to determine factors significantly associated with stress fracture risk (p < 0.05). Fracture locations were descriptively analyzed.

Results

The fracture group was younger (21.78 ± 3.70 vs 24.50 ± 3.33 years) and presented earlier in training (12.1 ± 1.2 days vs. 30.4 ± 22.5 days). They had higher rates of military shoe usage (70.5% vs. 23%), smoking history (64.03% vs. 20.4%), and lower vitamin D levels (26.63 ± 16.56 vs. 42.97 ± 13.75 ng/mL) (all p < 0.001). The most common fracture site was the tibial plateau (43.2%), and 23.0% of cases involved multiple sites. Most fractures (80.6%) were high-grade (Fredericson Grade 3 or 4).

Conclusion

In this retrospective case-control study, stress fracture occurrence among military trainees was significantly associated with younger age, the early phase of military training, vitamin D deficiency, smoking, use of standard-issue military footwear, and lack of prior physical training. These findings should be interpreted as associations and warrant confirmation in prospective studies with multivariable analyses.

## Introduction

Stress fractures are common and debilitating overuse injuries affecting athletes, military recruits, and individuals engaged in repetitive high-impact activities. These injuries occur when repetitive mechanical loading exceeds the bone's capacity for repair, leading to the accumulation of microdamage and eventual structural failure. The underlying pathophysiology involves an imbalance between bone resorption and formation, where osteoclastic activity outweighs osteoblastic activity [1]. Military trainees are particularly vulnerable, as they often experience abrupt transitions from sedentary lifestyles to rigorous physical training, placing excessive stress on bone structures that have not yet adapted to high mechanical demands [2].

A wide range of potential risk factors for stress fractures has been identified in the literature. These include demographic variables (e.g., age, sex), biomechanical influences (e.g., footwear type, training load), and metabolic conditions (e.g., vitamin D deficiency) [3-5]. However, findings across studies are inconsistent. Some studies suggest that older age increases fracture risk [6], while others report a higher incidence among younger individuals, particularly in contexts involving high-intensity training. Similarly, while female participants often exhibit higher stress fracture rates in athletic settings [7], male predominance has been observed in certain military cohorts, likely reflecting differences in training intensity, equipment usage, and behavioral factors such as smoking.

Understanding the multifactorial etiology of stress fractures is essential for developing effective prevention strategies in high-risk populations. Identifying both modifiable factors, such as nutritional status, smoking, training load, and footwear, and non-modifiable factors like age and prior injury can inform individualized risk assessments. In physically demanding environments like military training, early identification and targeted preventive measures are critical to reducing the burden of stress fractures and improving overall health and performance outcomes [8,9]. Stress fractures are prevalent in military populations due to repetitive loading, but many risk factors remain poorly defined or inconsistently supported across studies.

Therefore, the purpose of this retrospective case-control study was to identify and characterize the demographic, clinical, lifestyle, and training-related factors associated with stress fractures, as well as their anatomical distribution, among Jordanian military trainees. Specifically, this study aimed to answer the following research question: What demographic, clinical, lifestyle, and training-related factors are associated with stress fractures, and what is the anatomical distribution of stress fractures among Jordanian military trainees?

## Materials and methods

Study design

This retrospective case-control study was conducted at the Royal Medical Services of Jordan. Medical records of military trainees who presented with lower-limb pain during military training between January 2021 and June 2023 and underwent definitive imaging evaluation for suspected stress fractures were retrospectively reviewed. All eligible participants were drawn from this symptomatic, imaging-evaluated population and were subsequently classified according to their imaging findings into two groups: (1) trainees with radiologically confirmed stress fractures (cases) and (2) trainees with clinical suspicion of stress fractures but negative definitive imaging findings (controls). The study protocol was approved by the Institutional Ethics Committee of the Royal Medical Services, and all patient data were de-identified prior to analysis to ensure confidentiality.

Study population and inclusion criteria

This retrospective case-control study was conducted at the Royal Medical Services of Jordan. The study included military trainees who presented to King Hussein Medical City for evaluation of lower-limb pain during the training period between January 2021 and June 2023. A total of 600 consecutive eligible military trainees meeting the predefined inclusion criteria were included in the final analysis, comprising 139 trainees with radiologically confirmed stress fractures (cases) and 461 trainees without evidence of stress fractures on definitive imaging (controls). All consecutive eligible participants meeting the study criteria during the predefined study period were included.

Both cases and controls were derived from the same source population of military trainees presenting with lower-limb pain who underwent definitive imaging evaluation for suspected stress fractures during the study period.

Case Definition

Cases consisted of symptomatic military trainees who underwent definitive imaging evaluation for suspected stress fractures and had radiologically confirmed stress fractures on radiographs, MRI, or bone scan.

Control Definition

Controls consisted of trainees from the same training cycles who presented with clinical suspicion of a stress fracture but had no evidence of fracture on definitive imaging (MRI or bone scan).

Exclusion criteria applied to both groups included the following:

(1) Incomplete medical records, (2) known underlying metabolic bone disease (e.g., osteoporosis, osteomalacia), or (3) a recent acute traumatic fracture unrelated to repetitive stress.

Subject selection rationale

All consecutive eligible cases presenting within the study period were included. For each case, an attempt was made to identify multiple controls from the same training cycle to increase statistical power and control for temporal training variations. All military trainees had undergone a standard pre-enrollment medical screening and were deemed fit for service, providing a baseline healthy population.

Sample size rationale

As this was a retrospective case-control study, the sample size was determined by the number of consecutive eligible military trainees identified during the predefined study period (January 2021 to June 2023). Accordingly, all eligible cases meeting the inclusion criteria, together with their corresponding controls, were included to minimize selection bias and maximize the available study population. Because participant recruitment had already occurred before study initiation, a prospective a priori sample size calculation was not applicable. The final sample was considered adequate to evaluate the associations between potential risk factors and stress fracture occurrence among the included military trainees.

Data collection and variables

Demographic data (age, sex, height, weight), lifestyle factors (smoking history, prior regular physical activity), clinical variables (vitamin D levels, serum calcium, history of knee injuries), and training-related variables (type of footwear used, training duration before symptom onset) were collected through medical records and structured intake forms. The stress fracture site was documented through radiological reports and classified according to anatomical site. Cases with fractures at more than one anatomical site were recorded under a “multiple sites” category. Clinical data were documented according to the standards of the Jordanian Royal Medical Services. The study population was composed exclusively of Jordanian Arab military trainees.

Statistical analysis

Data were entered and analyzed using IBM SPSS Statistics for Windows, Version 26.0 (IBM Corp., Armonk, NY, USA). Normality of continuous variables was assessed using visual methods (histograms and Q-Q plots) and statistical tests including the Shapiro-Wilk and Kolmogorov-Smirnov tests. Variables with a normal distribution were expressed as mean ± standard deviation (SD), while non-normally distributed variables were presented as medians with interquartile ranges (IQR). Categorical variables were reported as frequencies and percentages. Group comparisons between cases and controls were performed using the Student’s t-test for normally distributed variables, the Mann-Whitney U test for skewed variables, and the chi-square test or Fisher’s exact test for categorical data. A two-sided p-value of <0.05 was considered statistically significant. Multivariable regression analysis was not performed as part of the original statistical analysis plan. Consequently, the reported findings represent unadjusted associations between the evaluated variables and stress fracture occurrence.

## Results

Demographic, clinical, and lifestyle factors associated with stress fractures

A total of 139 military trainees with confirmed stress fractures and 461 controls without stress fractures were included in the analysis. Participants with stress fractures were younger than controls (21.78 ± 3.70 vs. 24.50 ± 3.33 years, p = 0.03). There were no statistically significant differences between the groups in sex distribution (male participants: 64.0% vs. 57.1%; p = 0.143) or height (1.73 ± 0.05 m vs. 1.72 ± 0.07 m; p = 0.568). However, participants with stress fractures had a lower mean body weight (65.21 ± 9.71 kg vs. 67.45 ± 9.83 kg; p = 0.047). The mean duration of training prior to presentation was significantly shorter in the stress fracture group (12.1 ± 1.2 days vs. 30.4 ± 22.5 days; p < 0.001). Military shoe use was more common among participants with stress fractures (70.5% vs. 23.0%; p < 0.001), whereas a history of regular physical training before recruitment was less frequent (3.3% vs. 28.1%; p < 0.001). Participants with stress fractures had lower serum vitamin D levels (26.63 ± 16.56 ng/mL vs. 42.97 ± 13.75 ng/mL; p < 0.001) but higher serum calcium levels (9.70 ± 0.46 mg/dL vs. 9.43 ± 0.52 mg/dL; p < 0.001). Smoking was significantly more prevalent among participants with stress fractures (64.03% vs. 20.4%; p < 0.001), and a history of knee meniscal injury was observed only in the stress fracture group (2.16% vs. 0%; p < 0.001) (Table 1).

**Table 1:** Patient demographics Comparison of demographic, clinical, and training-related factors between military trainees with and without stress fractures. Continuous variables were compared using the independent-samples t-test or Mann-Whitney U test, as appropriate, while categorical variables were analyzed using the chi-square test or Fisher's exact test. Corresponding test statistics and p-values are reported.

Variable	Stress fracture (n = 139)	No stress fracture (n = 461)	Statistical test (Statistic)	p-Value
Age	21.78 (3.70)	24.50 (3.33)	t = 2.34	0.03
Gender			χ² = 2.41	0.143
Male	89 (64.0%)	263 (57.1%)		
Female	50 (36.0%)	198 (43.0%)		
Height (m) (mean)	1.73 (0.05)	1.72 (0.07)	χ² = 1.58	0.568
Weight (kg) (mean)	65.21 (9.71)	67.45 (9.83)	χ² = 1.62	0.047
Duration of training elapsed till time of presentation (days)	12.1 ± 1.2	30.4 ± 22.5	U = 0.547	<0.001
Usage of military shoe during training	98 (70.5%)	106 (23%)	χ² = 3.06	<0.001
Vitamin D level at the time of presentation (20-100 mg/dL)	26.63 (16.56)	42.97 (13.75)	U = 0.425	<0.001
Calcium level at the time of presentation (mg/dL)	9.70 (0.46)	9.43 (0.52)	U = 0.578	<0.001
History of smoking	89 (64.0%)	94 (20.4%)	χ² = 3.06	<0.001
History of knee meniscal injury	3 (2.2%)	0 (0%)	t = 1.25	<0.001
History of previous regular training before recruitment	15 (3.3%)	39 (28.1%)	U = 5721.0	<0.001

Anatomical distribution of stress fracture sites

**Figure 1:**
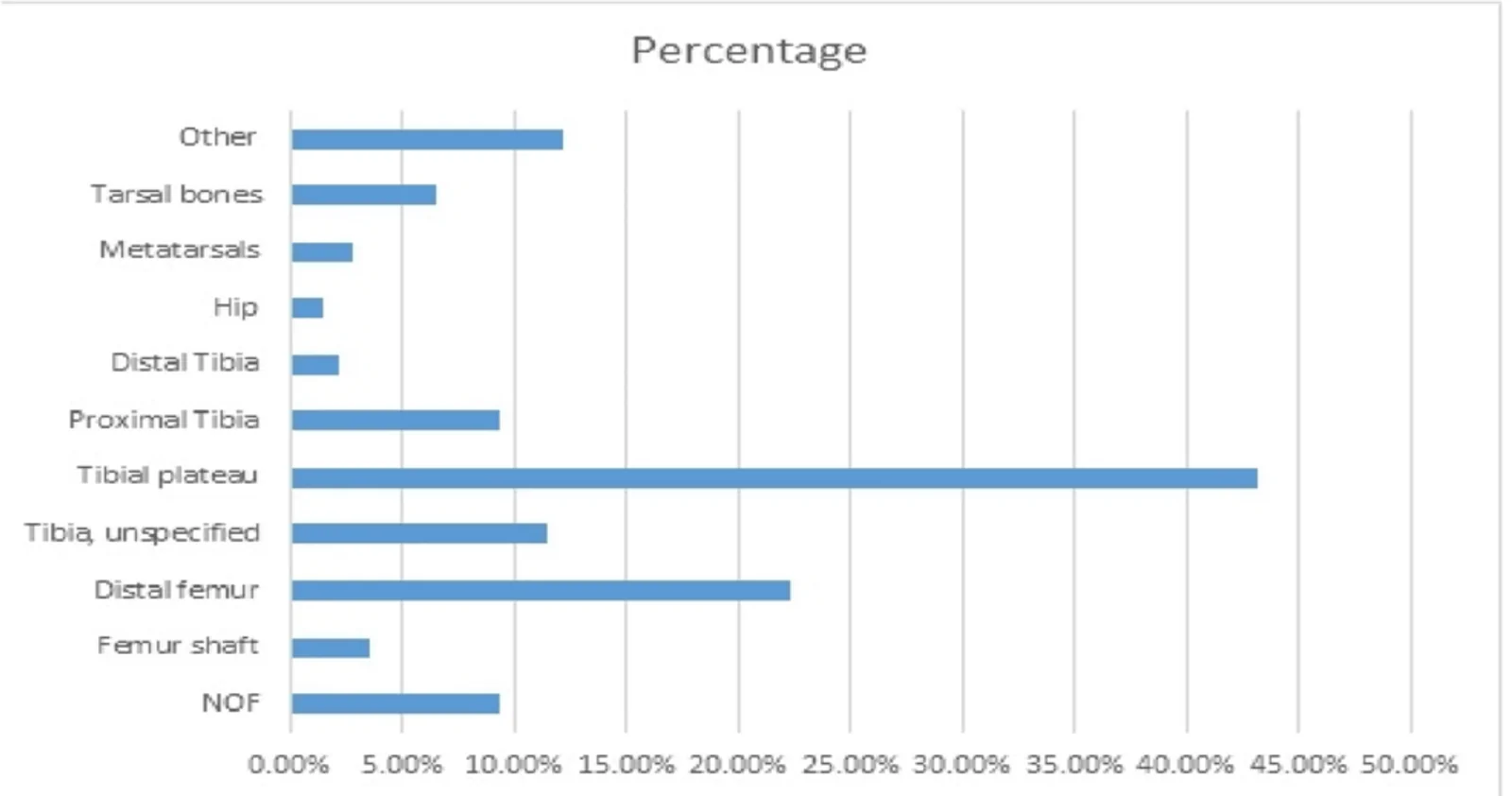
Distribution of stress fracture sites among affected individuals NOF, neck of femur.

**Figure 2:**
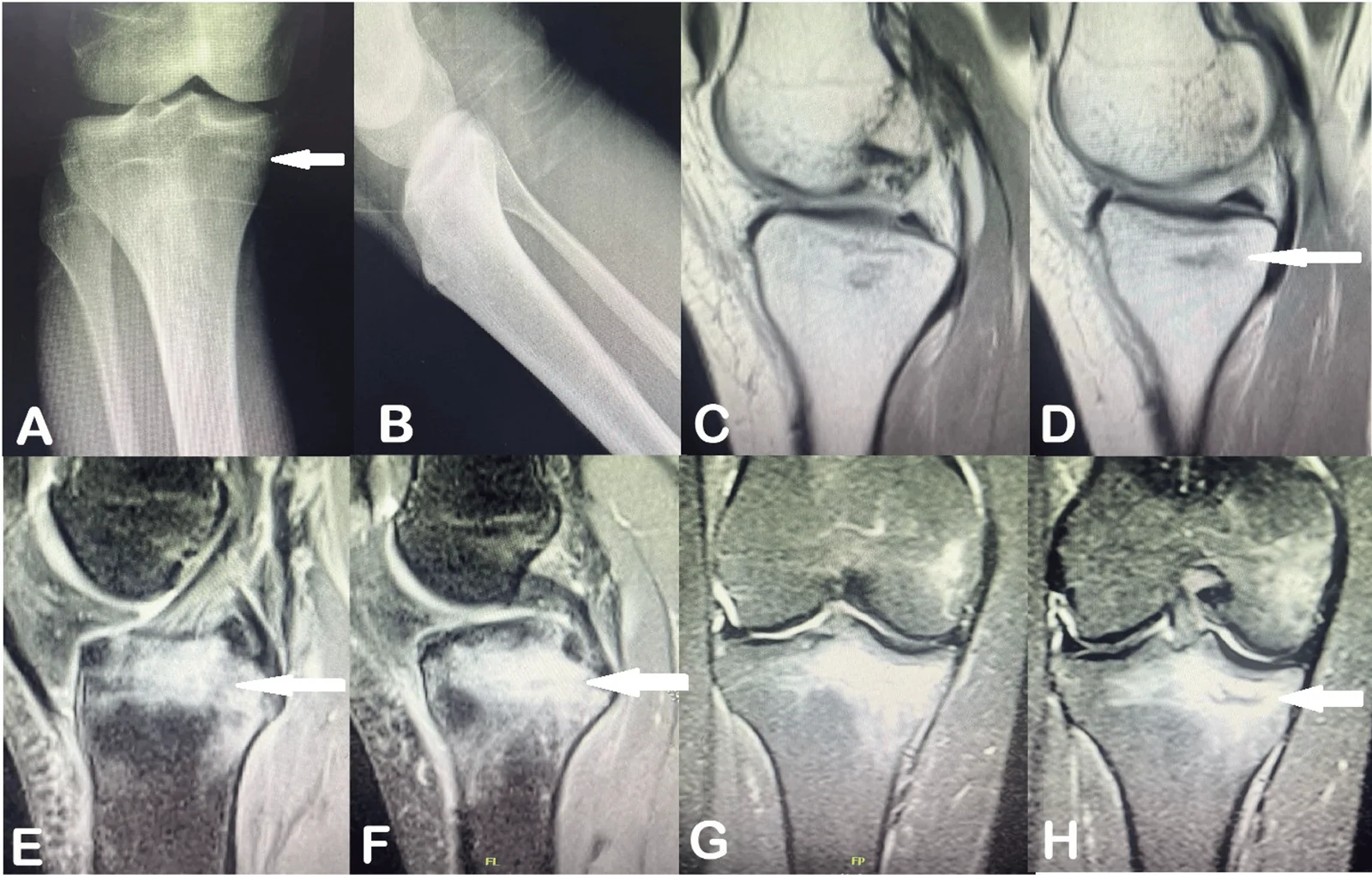
AP and lateral knee radiographs of a 26-year-old female (A, B) AP and lateral knee radiographs obtained at the initial presentation of a 26-year-old female military recruit showing no obvious pathology, with a possible stress fracture line (white arrow). (C, D) T1 sagittal knee MRI images of the same patient showing a fracture line of low signal without obvious signs of bone edema (white arrow). (E-H) T2 sagittal (E+F) and coronal (G+H) knee MRI images showing an obvious fracture line surrounded by significant bone edema representing a stress fracture (white arrow). AP, anteroposterior.

**Figure 3:**
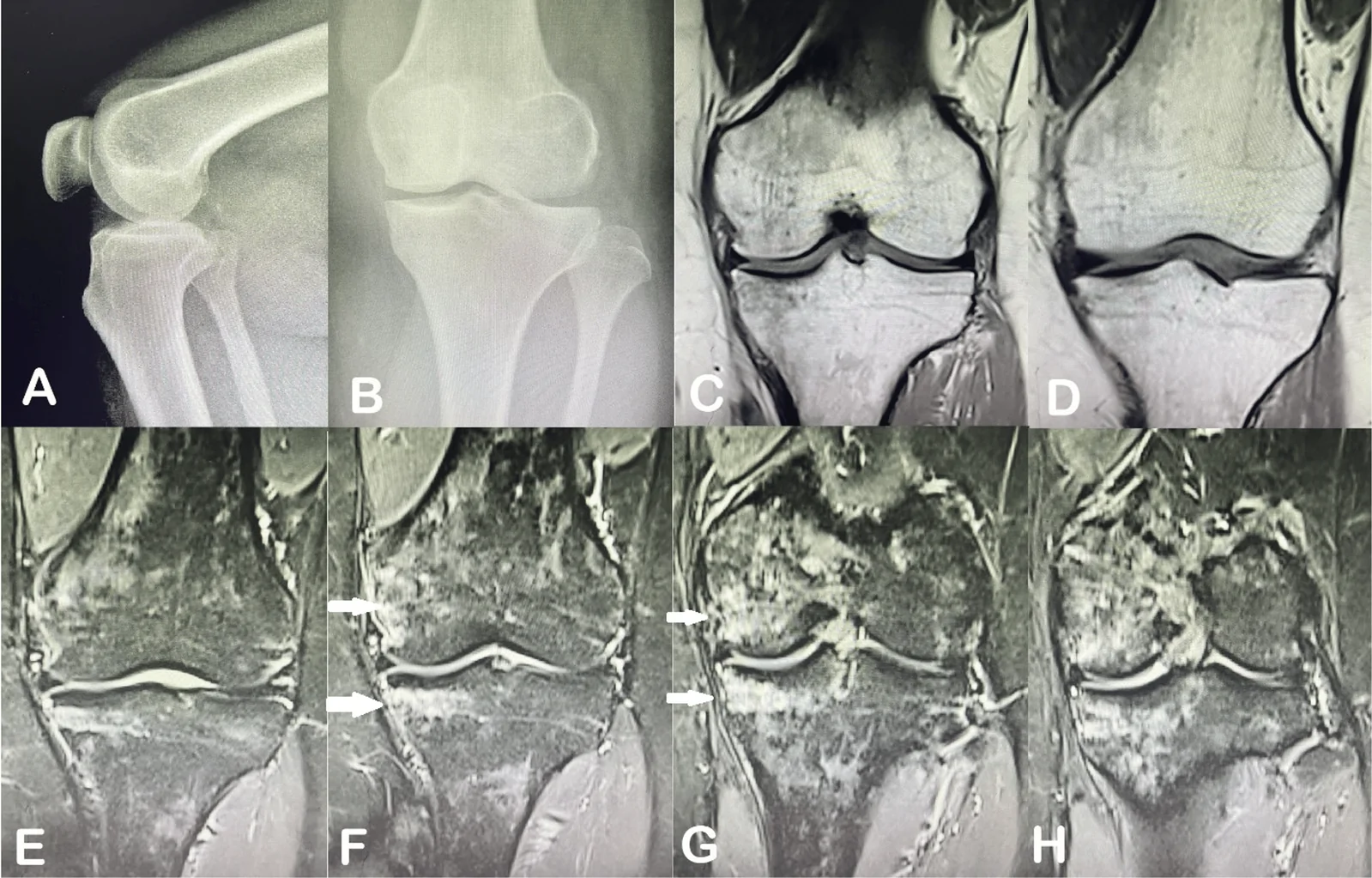
Lateral and AP left knee radiographs of a 27-year-old female (A, B) Lateral and AP left knee radiographs obtained at the initial presentation of a new 27-year-old female military recruit complaining of left knee pain, showing no obvious pathology. (C, D) T1 coronal knee MRI images of the same patient showing no obvious fracture line with signs of bone edema in the medial distal femur condyle and the medial tibial plateau. (E-H) T2 coronal knee MRI images showing obvious bone edema in the distal medial femur condyle and the medial tibial plateau representing a stress fracture (white arrow). AP, anteroposterior.

**Figure 4:**
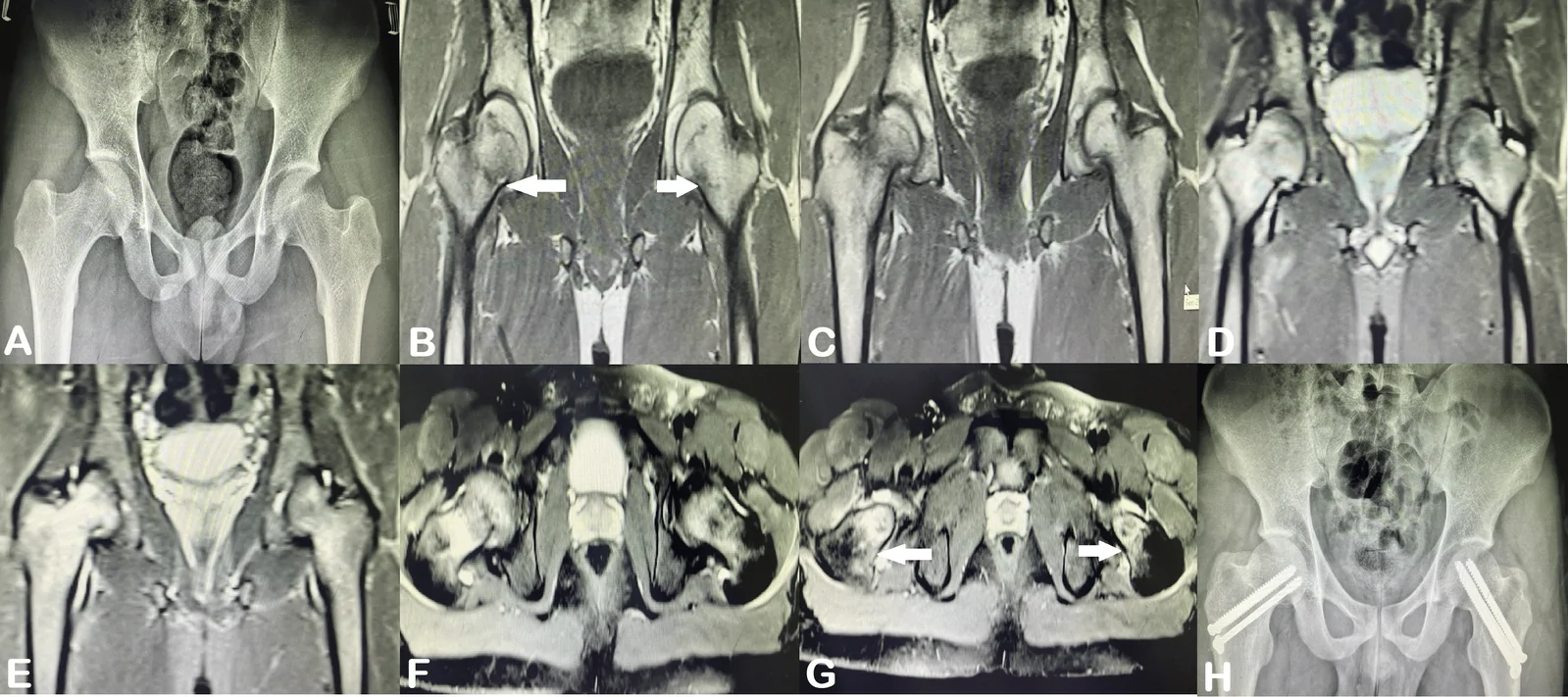
Pelvis radiograph of a 23-year-old male (A) AP pelvis radiograph obtained at the initial presentation of a new 23-year-old male military recruit complaining of bilateral hip pain, showing no obvious pathology. (B, C) T1 coronal bilateral hip MRI images of the same patient showing an obvious fracture line on the right neck of femur with signs of bone edema in the compression aspect of the left neck of femur (white arrow). (D, E) T2 coronal hip MRI images showing obvious bone edema in both necks of femur. (F, G) T2 axial MRI images of both hips showing no obvious fracture line on either side, with significant bilateral neck of femur bone edema representing bilateral neck of femur stress fracture (white arrow). (H) Postoperative two-month follow-up AP pelvis radiograph of the same patient showing the fixation of both fractures by cannulated screws without further displacement. AP, anteroposterior.

**Figure 5:**
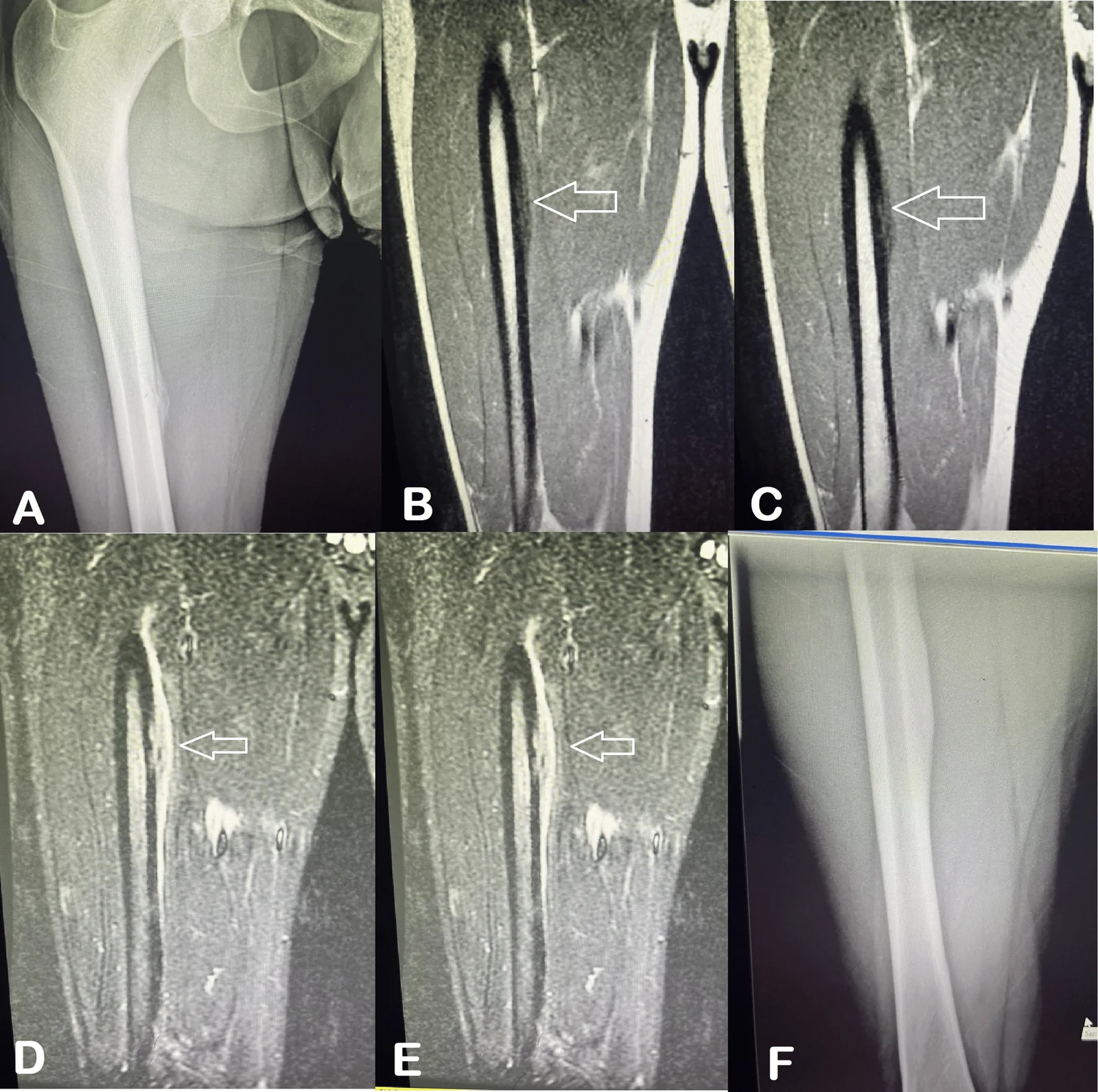
Femur radiograph of a 19-year-old male (A) AP femur radiograph obtained at the initial presentation of a new 19-year-old male military recruit complaining of right thigh pain, showing an incomplete stress fracture of the femur shaft on the compression side with callus formation. (B, C) T1 coronal thigh MRI images of the same patient showing periosteal reaction on the compression side of the femur without an obvious fracture line (arrow). (D, E) T2 coronal thigh MRI images showing obvious periosteal reaction with bone edema on the compression side of the femur shaft representing a femur stress fracture (arrow). (F) Three-month follow-up AP femur radiograph of the same patient showing complete healing after conservative treatment with complete resolution of symptoms. AP, anteroposterior.

**Figure 6:**
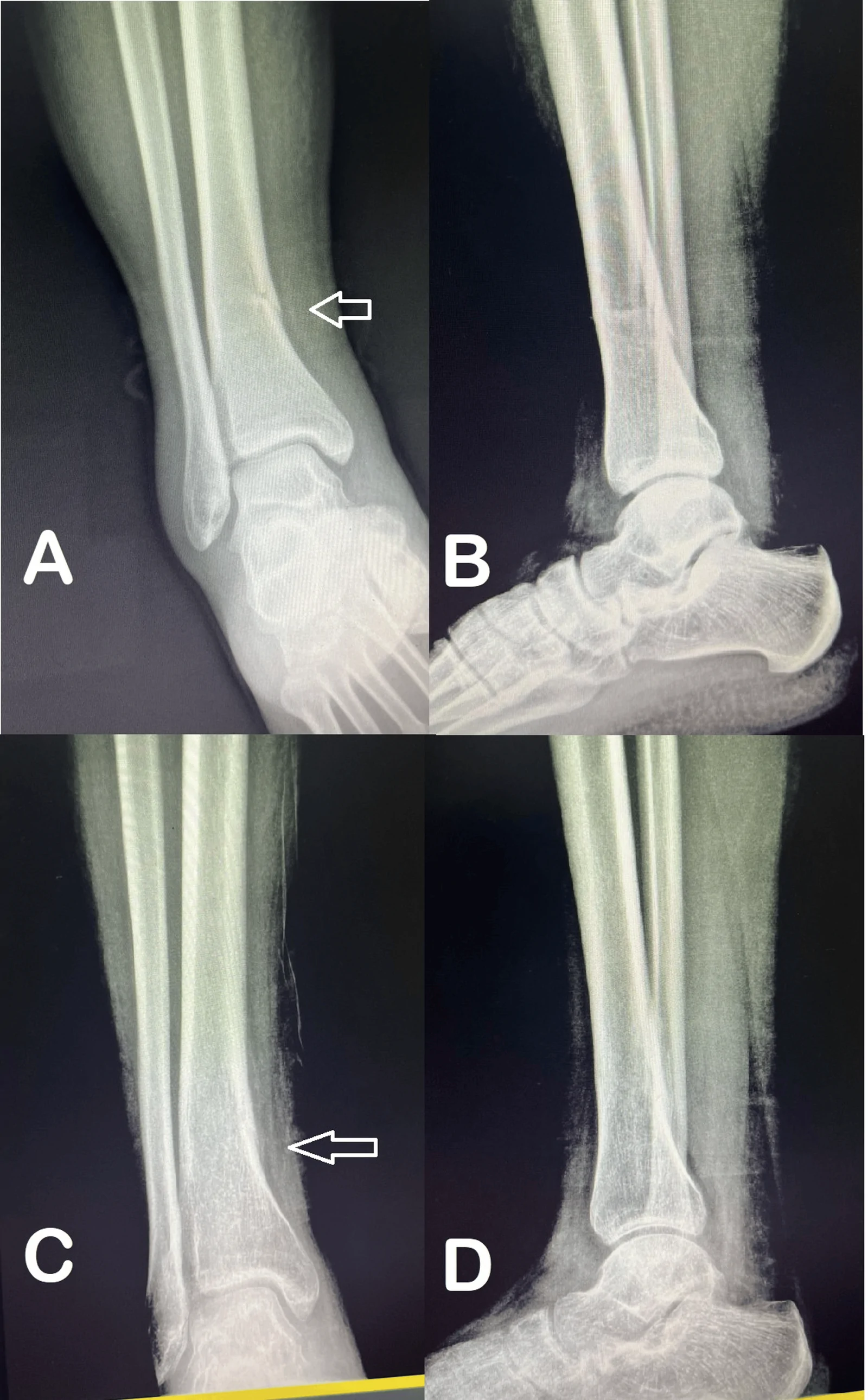
Leg radiographs of a 23-year-old male (A, B) AP and lateral distal leg radiographs obtained at the initial presentation of a 23-year-old male patient complaining of leg pain, showing an incomplete distal tibial stress fracture (arrow). (C, D) Three-month follow-up AP and lateral radiograph views of the same patient showing complete healing of the fracture, which was treated conservatively with a long leg cast, with complete resolution of the symptoms (arrow). AP, anteroposterior.

**Figure 7:**
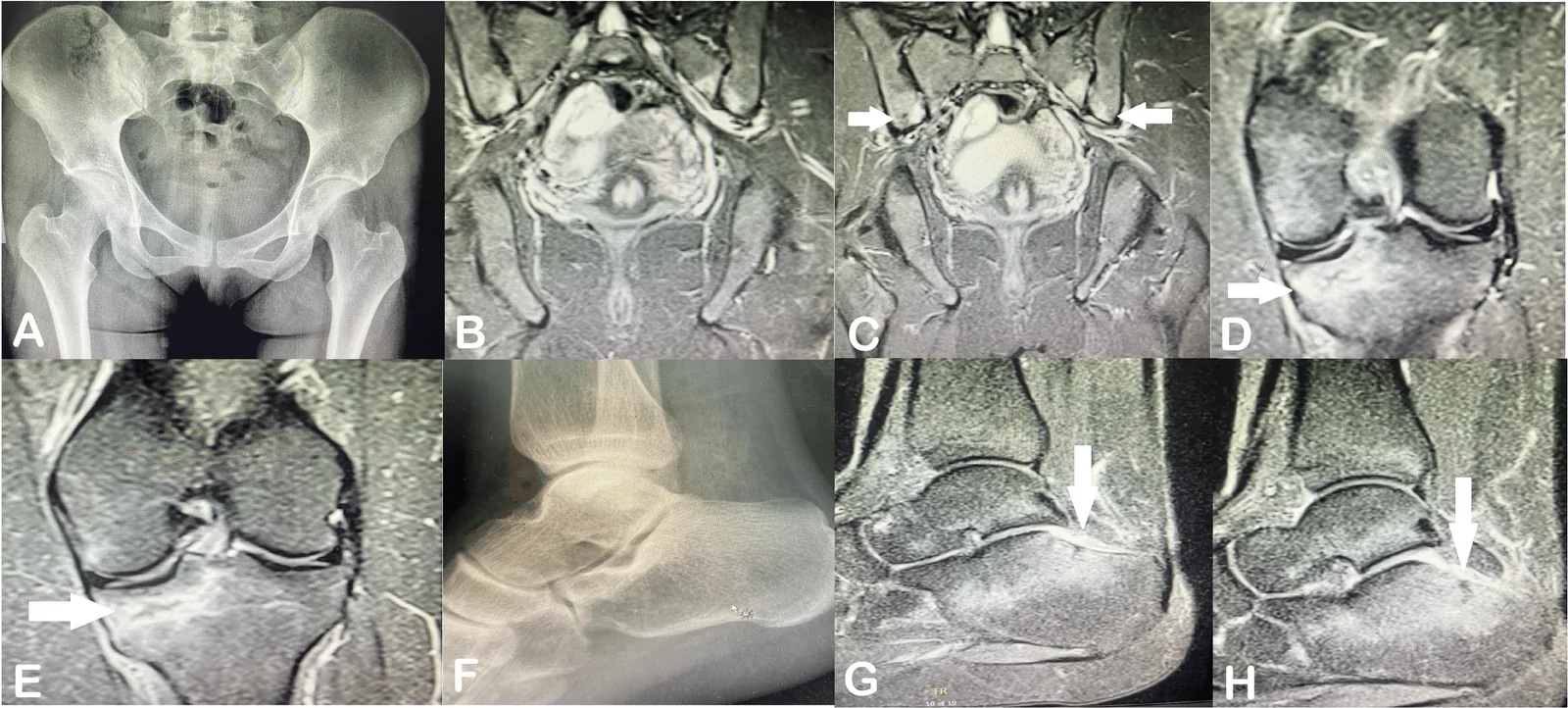
Pelvis radiograph of a 23-year-old female (A) AP pelvis radiograph obtained at the initial presentation of a new 23-year-old female military recruit complaining of left hip pain and right knee and right ankle pain, showing no obvious pathology. (B, C) T2 coronal pelvis MRI images of the same patient showing left sacroiliac bone edema representing a sacroiliac stress fracture (arrow). (D, E) T2 coronal knee MRI images of the same patient showing obvious medial tibial plateau bone edema with a fracture line representing a medial tibial plateau stress fracture (arrow). (F) Lateral ankle radiograph obtained at the initial presentation of the same patient showing no obvious pathology. (G, H) T2 sagittal ankle MRI images of the same patient showing distal tibia, talus, and calcaneus bone edema without a fracture line representing multiple stress fractures (arrow). AP, anteroposterior.

The distribution of stress fracture locations revealed varied patterns among the 139 patients (Figure 1). The most common site was the tibial plateau (n = 60, 43.2%; Figure 2), followed by the distal femur (n = 31, 22.3%; Figure 3) and tibia, unspecified (n = 16, 11.51%). Less commonly, fractures of the proximal tibia (n = 13, 9.35%), neck of femur (n = 12, 9.35%; Figure 4), and tarsal bones (n = 9, 6.47%) were identified. Fractures of the femur shaft (n = 5, 3.60%; Figure 5), metatarsals (n = 4, 2.88%), distal tibia (n = 2, 1.44%; Figure 6), and hip (n = 2, 1.44%) were the least common. Other sites accounted for 12.23% (n = 17) of cases. Thirty-two cases (23.02%) had multiple sites of fracture (Figure 7; Table 2).

**Table 2:** Anatomical location distribution Distribution of stress fracture sites among affected individuals. NOF, neck of femur.

Site of fracture	Number	Percentage
NOF	13	9.4
Femur shaft	5	3.6
Distal femur	31	22.3
Tibia, unspecified	16	11.5
Tibial plateau	60	43.2
Proximal tibia	13	9.4
Distal tibia	3	2.2
Hip	2	1.4
Metatarsals	4	2.9
Tarsal bones	9	6.5
Other	17	12.2
Multiple sites	32	23.0

Severity of stress fractures according to Fredericson classification

The severity of the 139 radiologically confirmed stress fractures was graded using the Fredericson MRI classification system (Table 3) [10]. Fractures were distributed across all Fredericson grades: 9 (6.5%) were Grade 1 (mild bone marrow edema (BME)), 18 (12.9%) were Grade 2 (moderate-to-severe BME), 25 (18%) were Grade 3 (fracture line without cortical breach), 23 (17%) were Grade 4a (fracture line with periosteal edema), and 64 (46%) were Grade 4b (fracture line with full-thickness cortical involvement). In total, the majority of injuries (112/139, 80.6%) were high-grade (Grade 3, 4a, or 4b), confirming the presence of a discrete fracture line, with a substantial proportion (87/139, 62.6%) demonstrating cortical involvement (Grade 4).

**Table 3:** Severity of cases Severity of stress fractures according to Fredericson MRI classification among affected individuals [10].

Fredericson grade	1	2	3	4a	4b
Number of male patients	2	5	10	13	20
Number of female patients	7	13	15	10	44

## Discussion

The findings of this retrospective case-control study of Jordanian military trainees demonstrate that stress fractures are associated with a multifactorial risk profile. Key risk factors include younger age, earlier presentation during training, vitamin D deficiency, smoking, use of standard-issue military footwear, lack of prior conditioning, and a history of knee meniscal injury. The tibial plateau was the most common site of injury, and a high proportion of fractures were severe (high-grade). These results underscore the complex interplay of demographic, metabolic, behavioral, and equipment-related factors in the development of bone stress injuries in this population.

Stress fractures represent a significant health concern among young adults, particularly in athletic and military populations. Stress fractures are caused by an imbalance between bone resorption and bone formation. This occurs due to a dominance of osteoclast activity over osteoblast activity [11]. These injuries occur when repetitive mechanical loading exceeds the bone's ability to repair microdamage, leading to structural weakness and potential fracture [12].

The observed association between younger age and stress fractures among Jordanian military trainees contrasts with some previous studies suggesting older age as a risk factor [13]. This discrepancy may reflect the unique physical demands of military training, where younger recruits often undergo abrupt transitions to high-intensity activity without adequate musculoskeletal conditioning [14].

This study demonstrated a higher proportion of stress fractures among male participants, contrasting with much of the existing literature, which generally reports a higher incidence among female participants [15]. The observed male predominance in the present study may reflect the characteristics of the study population, including the higher proportion of male military recruits, training-related factors such as heavy load carriage and use of standard-issue military footwear, and behavioral factors including the higher prevalence of smoking among male participants. Although the difference in sex distribution was not statistically significant, these findings suggest that the epidemiology of stress fractures may vary according to the study population and training environment. Military training, characterized by abrupt increases in physical demands and equipment-related factors, may produce a different pattern of stress fracture occurrence compared with athletic populations, where biological factors have traditionally been considered more influential [7]. These observations underscore the importance of population-specific risk assessment rather than generalizing findings across different settings.

The lower weight observed in the stress fracture group further supports the hypothesis that inadequate musculoskeletal adaptation to mechanical loads plays a critical role and aligns with previous studies indicating the relation between weight and stress fractures [16]. These individuals may lack the muscle mass necessary to absorb and distribute forces effectively, increasing bone strain.

The data show a significant difference in duration of training elapsed until presentation between stress fracture cases and non-cases, suggesting that stress fractures occurred early in training, likely due to abrupt increases in activity intensity without adequate bone adaptation. The rapid onset of debilitating symptoms, prompting medical consultation within days, occurred in trainees who were largely unconditioned prior to recruitment. Although the initial clinical presentation was early, the subsequent radiological confirmation via MRI and bone scan -- typically scheduled two to four weeks later -- provided the necessary interval for the classic imaging features of stress fractures to become fully apparent. This aligns with existing literature identifying sudden training load escalation as a key risk factor, particularly in military populations, where rapid transitions from sedentary lifestyles to high-impact drills overwhelm bone remodeling capacity [17]. This underscores the importance of gradual conditioning programs, as shorter adaptation periods correlate with higher stress fracture incidence.

Vitamin D deficiency emerged as a significant risk factor, consistent with its well-documented role in bone health [18]. Lower vitamin D levels impair calcium absorption and bone mineralization, rendering bones more susceptible to microfractures under repetitive stress. The higher calcium levels in the stress fracture group, while seemingly counterintuitive, may reflect compensatory mechanisms or differences in dietary intake [19].

In contrast, although serum calcium levels were statistically higher in the stress fracture group, the absolute difference between groups was small (9.70 vs. 9.43 mg/dL) and remained within the normal physiological range. Therefore, the clinical significance of this finding should be interpreted cautiously. The observed difference may reflect normal biological variation, dietary factors, hydration status, or other unmeasured influences rather than a clinically meaningful determinant of stress fracture occurrence. Accordingly, serum calcium alone is unlikely to have substantial clinical relevance as an isolated factor associated with stress fracture occurrence and should be interpreted in conjunction with other clinical and metabolic factors.

The data revealed a significant association between smoking and stress fracture occurrence. This finding is consistent with previous studies demonstrating the adverse effects of smoking on bone health and its association with stress fractures [16,20,21]. Smoking has been shown to impair bone metabolism through multiple mechanisms, including reduced osteoblast activity, increased bone resorption, impaired calcium absorption, diminished peripheral blood supply, and delayed bone remodeling, all of which may compromise the repair of repetitive microdamage and increase susceptibility to stress fractures [21]. The marked difference in smoking prevalence between the two groups underscores the importance of smoking cessation as a potentially modifiable factor for reducing stress fracture occurrence among military trainees. Further research is warranted to better elucidate the biological mechanisms linking smoking, bone remodeling, and stress fracture occurrence and to evaluate the impact of smoking cessation on injury prevention in high-risk military populations [17].

Military shoe use during training was significantly associated with stress fracture occurrence in the present study. However, this finding should be interpreted cautiously because footwear use is likely influenced by other factors, including training intensity, cumulative mechanical loading, previous physical conditioning, and other participant characteristics that were not simultaneously adjusted for in the present analysis. Consequently, the observed association should be considered hypothesis-generating rather than evidence of a direct causal relationship. Nevertheless, previous studies have suggested that footwear characteristics, including shock absorption and biomechanical support, may influence lower-limb loading during repetitive physical activity [22,23]. Therefore, further prospective studies incorporating multivariable analyses and detailed biomechanical assessment of military footwear are warranted before specific recommendations regarding standard-issue footwear can be made.

The exclusive presence of knee meniscal injury in the stress fracture group underscores the importance of prior musculoskeletal injuries as predisposing factors and aligns with existing literature suggesting that prior musculoskeletal injuries, particularly those affecting load-bearing joints, may predispose individuals to stress fractures due to altered biomechanics, compensatory overloading, or incomplete rehabilitation [24,25].

The distribution of stress fracture sites reveals that the tibial plateau was the most frequently affected location, followed by the distal femur. This pattern aligns with existing literature, as the tibia is a common site for stress fractures due to its high load-bearing role during weight-bearing activities [26]. The prevalence of multiple-site fractures suggests significant biomechanical stress or systemic risk factors (e.g., low vitamin D, as noted in Table 1). Notably, metatarsals and tarsal bones were less frequent (2.9% and 6.5%, respectively), which may reflect differences in training surfaces or footwear (e.g., military shoes, which were heavily associated with fractures in Table 1). The high proportion of tibial and femoral fractures underscores the need for targeted preventive measures, such as gait analysis, load management, and nutritional interventions, particularly in populations with the risk factors identified in Table 1 (e.g., smoking, low vitamin D).

MRI assessment in the present study demonstrated that radiologically confirmed stress fractures consistently occurred at a characteristic distance from the joint line, approximately at the level of the epiphyseal plate. This observation is consistent with the findings of Yukata et al. [27], who described reproducible stress fracture localization within the medial tibial plateau related to tibial slope morphology, suggesting that concentrated mechanical loading at the metaphyseal-diaphyseal transition may contribute to fracture development. The severity of radiologically confirmed stress fractures was graded using the Fredericson MRI classification, a validated imaging-based system in which Grade 1 represents mild BME, Grade 2 moderate-to-severe BME, Grade 3 a fracture line without cortical breach, and Grade 4 a fracture line with cortical involvement (Grade 4a: periosteal; Grade 4b: full-thickness) [10]. The distribution of Fredericson grades in the present study is shown in Table 3. Most injuries (112/139, 80.6%) were classified as high-grade (Grades 3 or 4), confirming the presence of a discrete fracture line, while 64 cases (46.0%) were classified as Grade 4b, indicating full-thickness cortical involvement. This high proportion of advanced injuries may reflect delayed clinical presentation or diagnosis, continued exposure to repetitive mechanical loading during military training, or both, allowing bone stress injuries to progress before definitive imaging and diagnosis.

The predominance of high-grade (Fredericson Grades 3-4) stress fractures observed in the present study likely reflects the diagnostic pathway within our military healthcare system rather than delayed symptom reporting by recruits. Although trainees generally undergo prompt clinical assessment after developing persistent lower-limb pain, definitive imaging with MRI or bone scintigraphy is performed as a scheduled investigation rather than an emergency examination. Consequently, recruits often continue military training while awaiting advanced imaging, resulting in ongoing repetitive mechanical loading that may allow early bone stress injuries to progress to higher-grade fractures with visible fracture lines. These findings underscore the importance of timely recognition of suspected stress fractures and suggest that earlier access to advanced imaging, temporary activity modification, and standardized triage protocols for symptomatic recruits may facilitate diagnosis at an earlier stage and potentially reduce progression to more severe injuries.

Limitations

This study has several limitations that should be considered when interpreting the findings. First, as a retrospective case-control study, the sample size was determined by the number of eligible participants identified during the study period rather than by a prospective a priori power calculation. Furthermore, because of the retrospective design and the absence of multivariable regression analysis, the identified variables should be interpreted as factors associated with stress fracture occurrence rather than independent causal risk factors. The study population was limited to military trainees, restricting the generalizability of the findings to other populations such as civilian athletes, the general female population, or older adults, who may have different stress fracture risk profiles. Additionally, both cases and controls were derived from the same population of military trainees presenting with lower-limb pain who underwent definitive imaging evaluation for suspected stress fractures. Consequently, the control group represented a symptomatic, higher-risk population rather than asymptomatic military trainees. This sampling strategy may have introduced selection bias and may limit the generalizability of the observed associations to the broader military trainee population. Therefore, the findings should be interpreted within the context of symptomatic trainees undergoing diagnostic evaluation for suspected stress fractures. Moreover, the study population was predominantly male, reflecting military recruitment patterns, and may not accurately represent the sex distribution reported in other athletic or civilian populations. Finally, the relatively small size of certain subgroups, such as participants with a history of knee meniscal injury, limited the statistical power to evaluate these associations.

Several potentially important variables, including dietary habits, genetic predisposition, bone mineral density, training surfaces, and detailed exercise regimens, were not available in the medical records and therefore could not be evaluated. Similarly, the assessment of footwear lacked sufficient detail regarding shoe characteristics, wear patterns, and individual biomechanical factors, limiting the ability to formulate specific preventive recommendations. Consequently, some of the observed associations may have been influenced by residual confounding from unmeasured variables.

Finally, this study focused primarily on injuries occurring during the early phase of military training and therefore did not evaluate long-term clinical outcomes or the effectiveness of preventive interventions over time. Nevertheless, the observation that most stress fractures occurred within the first two weeks of training highlights the importance of gradual conditioning programs and individualized pre-training risk assessment. Future prospective studies incorporating multivariable analyses, longer follow-up, biomechanical assessments, and intervention strategies, such as optimized military footwear, vitamin D supplementation, smoking cessation, and structured injury-prevention programs, are warranted to better define independent risk factors and evaluate preventive measures. Importantly, the findings of the present study have already informed preventive initiatives within our institution, including the evaluation of improved military footwear and the implementation of targeted smoking cessation programs for incoming recruits.

## Conclusions

This retrospective case-control study identified several demographic, clinical, lifestyle, and training-related factors that were significantly associated with stress fracture occurrence among military trainees. Younger age, lower body weight, vitamin D deficiency, smoking, lack of prior physical conditioning, use of standard-issue military footwear, and a history of knee meniscal injury were significantly associated with stress fracture occurrence. The tibial plateau was the most frequently affected anatomical site, and most injuries were classified as high-grade stress fractures. These findings suggest that stress fracture occurrence among military trainees is influenced by multiple demographic, metabolic, behavioral, and training-related factors. However, because of the retrospective case-control design and the absence of multivariable adjustment, these findings should be interpreted as unadjusted associations rather than independent causal relationships. Future prospective studies incorporating multivariable analyses are warranted to confirm these associations and better define their independent contributions to stress fracture occurrence.

## References

[1] Warden SJ, Davis IS, Fredericson M (2014). Management and prevention of bone stress injuries in long-distance runners. J Orthop Sports Phys Ther.

[2] Dash N, Kushwaha A (2012). Stress fractures-A prospective study amongst recruits. Med J Armed Forces India.

[3] Knapik JJ, Trone DW, Tchandja J, Jones BH (2014). Injury-reduction effectiveness of prescribing running shoes on the basis of foot arch height: Summary of military investigations. J Orthop Sports Phys Ther.

[4] Lappe J, Cullen D, Haynatzki G, Recker R, Ahlf R, Thompson K (2008). Calcium and vitamin d supplementation decreases incidence of stress fractures in female navy recruits. J Bone Miner Res.

[5] Tenforde AS, Sayres LC, Sainani KL, Fredericson M (2010). Evaluating the relationship of calcium and vitamin D in the prevention of stress fracture injuries in the young athlete: A review of the literature. PM R.

[6] Knapik J, Montain SJ, McGraw S, Grier T, Ely M, Jones BH (2012). Stress fracture risk factors in basic combat training. Int J Sports Med.

[7] Wentz L, Liu PY, Haymes E, Ilich JZ (2011). Females have a greater incidence of stress fractures than males in both military and athletic populations: A systemic review. Mil Med.

[8] Abbott A, Bird ML, Wild E, Brown SM, Stewart G, Mulcahey MK (2020). Part I: Epidemiology and risk factors for stress fractures in female athletes. Phys Sportsmed.

[9] Knapik JJ, Reynolds KL, Hoedebecke KL (2017). Stress fractures: Etiology, epidemiology, diagnosis, treatment, and prevention. J Spec Oper Med.

[10] Fredericson M, Bergman AG, Hoffman KL, Dillingham MS (1995). Tibial stress reaction in runners. Correlation of clinical symptoms and scintigraphy with a new magnetic resonance imaging grading system. Am J Sports Med.

[11] McInnis KC, Ramey LN (2016). High-risk stress fractures: Diagnosis and management. PM R.

[12] Beck B, Drysdale L (2021). Risk factors, diagnosis and management of bone stress injuries in adolescent athletes: A narrative review. Sports (Basel).

[13] Kale NN, Wang CX, Wu VJ, Miskimin C, Mulcahey MK (2022). Age and female sex are important risk factors for stress fractures: A nationwide database analysis. Sports Health.

[14] Mann G, Hetsroni I, Constantini N (2015). Stress fractures: Introduction, risk factors, and distribution. Sports Injuries: Prevention, Diagnosis, Treatment and Rehabilitation.

[15] Beck TJ, Ruff CB, Shaffer RA, Betsinger K, Trone DW, Brodine SK (2000). Stress fracture in military recruits: Gender differences in muscle and bone susceptibility factors. Bone.

[16] Zalneraitis BH, Huuki E, Benavides LC, Benavides JM (2023). Relation of vitamin D level, BMI, and location of lower extremity stress fractures in military trainees. Mil Med.

[17] Jones BH, Thacker SB, Gilchrist J, Kimsey CD Jr, Sosin DM (2002). Prevention of lower extremity stress fractures in athletes and soldiers: A systematic review. Epidemiol Rev.

[18] Knechtle B, Jastrzębski Z, Hill L, Nikolaidis PT (2021). Vitamin D and stress fractures in sport: Preventive and therapeutic measures-A narrative review. Medicina (Kaunas).

[19] Sonneville KR, Gordon CM, Kocher MS, Pierce LM, Ramappa A, Field AE (2012). Vitamin D, calcium, and dairy intakes and stress fractures among female adolescents. Arch Pediatr Adolesc Med.

[20] Nieves JW, Melsop K, Curtis M (2010). Nutritional factors that influence change in bone density and stress fracture risk among young female cross-country runners. PM R.

[21] Wong PK, Christie JJ, Wark JD (2007). The effects of smoking on bone health. Clin Sci (Lond).

[22] Milgrom C, Giladi M, Kashtan H (1985). A prospective study of the effect of a shock-absorbing orthotic device on the incidence of stress fractures in military recruits. Foot Ankle.

[23] Ryan LM, Teach SJ, Singer SA (2012). Bone mineral density and vitamin D status among African American children with forearm fractures. Pediatrics.

[24] May H, Murphy S, Khaw KT (1995). Bone mineral density and its relationship to skin colour in Caucasian females. Eur J Clin Invest.

[25] Zhou T (2018). Analysis of the biomechanical characteristics of the knee joint with a meniscus injury. Healthc Technol Lett.

[26] Matheson GO, Clement DB, McKenzie DC, Taunton JE, Lloyd-Smith DR, MacIntyre JG (1987). Stress fractures in athletes. A study of 320 cases. Am J Sports Med.

[27] Yukata K, Yamanaka I, Ueda Y, Nakai S, Ogasa H, Oishi Y, Hamawaki JI (2017). Medial tibial plateau morphology and stress fracture location: A magnetic resonance imaging study. World J Orthop.

